# Electrospun PVA nanofibers improve the storage stability of *Bacillus amyloliquefaciens* fermentation broth

**DOI:** 10.1007/s11274-026-05334-w

**Published:** 2026-10-09

**Authors:** Paula Derksen Macruz, Giovanna Picoli Libel, Lucas Haruo Nakayama Pereira, Juliano Cesar da Silva, Sergio Antônio Barraca, Eduardo Radovanovic, Eduardo Jorge Pilau

**Affiliations:** 1 Department of Chemical, State University of Maringá (UEM), Maringá, Paraná Brazil; 2 Postgraduate Program in Biological Sciences, State University of Maringá (UEM), Maringá, Paraná Brazil; 3 Ballagro Agro Tecnologia, Sumaré, SP Brazil

**Keywords:** Microbial formulation, *Bacillus amyloliquefaciens*, Electrospinning, Storage stability, Microbial viability, PVA nanofibers

## Abstract

The development of robust, stable microbial formulations remains a major challenge for agricultural use. We report an electrospinning-based formulation that encapsulates *Bacillus amyloliquefaciens* fermentation broth within poly(vinyl alcohol) (PVA) nanofibers to enhance storage stability and preserve microbial viability. Electrospun nanofiber mats were obtained from PVA formulations containing the fermentation broth, and their morphology, chemical profile, mechanical properties, and microbial viability were evaluated. Morphological, spectroscopic, mechanical, and microbiological analyses demonstrated successful incorporation of the fermentation broth into the PVA matrix and retention of viable *Bacillus* cells after electrospinning, with comparative estimates of viable cell recovery of 3.3** × **10^7^ CFU cm^− 2^ in the electrospun fibers and 8.3 × 10^7^ CFU mL^− 1^ in the polymeric spinning solution before electrospinning. Nanofibers stored under ambient conditions for up to one year still yielded viable cells. Collectively, these findings support the potential of electrospun PVA nanofibers as a formulation strategy for microbial stabilization and warrant further investigation toward the development of microbial formulations with improved storage characteristics.

**Supplementary Information:**

The online version contains supplementary material available at https://doi.org/10.1007/s11274-026-05334-w.

## Introduction

The adoption of microbial inoculants, such as *B. amyloliquefaciens*, has expanded owing to their proven ability to suppress phytopathogens, enhance nutrient uptake, and promote overall plant health (Luo et al. 2022). However, large-scale agricultural applications of microbial products face significant formulation challenges, including short shelf life, reduced microbial stability during storage, and sensitivity to environmental conditions during handling and application. These constraints limit product scalability and efficacy across diverse cropping systems (Luan et al. 2023).

To overcome these barriers, innovative encapsulation approaches are being investigated to improve microbial stability and formulation performance (Farias et al. 2019). Electrospinning has emerged as a promising technique for producing ultrafine polymeric fibers capable of encapsulating sensitive biological materials (Naeim et al. 2025). Electrospun nanofibers provide a high surface area-to-volume ratio, controlled porosity, and tunable structural and functional properties - characteristics extensively explored in biomedical contexts and increasingly investigated for microbial formulations for agricultural use (Shelar et al. 2023; Wang et al. 2025; Adhithiya Rajah et al. 2025).

Recent studies have demonstrated the feasibility of electrospun polymeric systems for agricultural use, particularly for preserving beneficial microorganisms within polymeric matrices. For example, plasticized electrospun biocomposites seed coats have been reported to improve microbial survival and formulation stability under controlled and semi-field conditions (Hussain et al. 2019). These findings highlight the potential of electrospinning-based platforms to overcome some of the limitations associated with conventional microbial carriers, such as rapid desiccation, uneven distribution, and insufficient retention of viable cells.

Beyond functional performance, electrospun bioformulations may also offer practical advantages for the storage and handling of microbial products. By improving microbial stability and persistence, such systems may reduce the need for cold-chain storage and help maintain formulation integrity over time. Moreover, the electrospinning process may improve the protection and shelf life of microbial formulations (Jurić et al. 2023). The use of water-soluble and widely used polymers, such as PVA or chitosan, ensures that the approach adheres to green chemistry and environmental safety standards (Soares et al. 2020).

Despite these advances, significant gaps persist in the development of electrospun microbial formulations suitable for agricultural use. Existing electrospinning-based approaches generally rely on time-consuming and costly downstream purification steps prior to electrospinning. For example, recent studies involving *Bacillus* and *Pseudomonas* strains require optical density adjustment of isolated cells (Pramparo et al. 2025) or extensive centrifugation, buffer washing, and biomass lyophilization before electrospinning (Vehapi et al. 2024). Likewise, electrospinning-based fungal biocontrol studies have required centrifugation to separate spores from the culture supernatant (Spasova et al. 2011). These labor-intensive procedures have largely overlooked an alternative strategy based on the direct incorporation of crude fermentation broth into electrospun matrices, thereby preserving the native microbial metabolites and biosurfactants naturally present in the fermentation medium.

Accordingly, the present study addresses this gap by directly electrospinning crude *B. amyloliquefaciens* fermentation broth without prior cell isolation, washing, or lyophilization. We hypothesize that the metabolites and other bioactive components naturally present in the fermentation broth may facilitate the electrospinning process, contribute to the mechanical properties of the resulting fibers, and create a protective microenvironment that helps preserve bacterial viability during electrospinning and prolonged ambient storage.

## Materials and methods

### Materials

*B. amyloliquefaciens* (CCT 7901) fermentation broth was kindly provided by Ballagro (São Paulo, Brazil). Polyvinyl alcohol (PVA; Mw 146,000–186,000, 87–89% hydrolyzed; Sigma-Aldrich) was used for preparation of the electrospinning solutions. Trypticasein soy agar (TSA K25-1068) was supplied by KASVI (Paraná, Brazil) and was used to prepare the culture medium.

### Electrospinning

Three electrospinning solutions were prepared in this study. The control PVA solution consisted of 12% (w/v) PVA prepared by dissolving 1.2 g of PVA in 10 mL of distilled water. For ease of reference, the PVA formulations containing *B. amyloliquefaciens* fermentation broth are referred to as PVA-BA50 and PVA-BA100 throughout the manuscript. The PVA-BA100 formulation consisted of 1.2 g of PVA dissolved in 10 mL of *B. amyloliquefaciens* fermentation broth, which served as the sole solvent. The PVA-BA50 formulation consisted of 1.2 g of PVA dissolved in a liquid phase composed of 5 mL of fermentation broth and 5 mL of sterile distilled water.

The mixtures were stirred at 50 °C for 2 h to ensure complete dissolution and homogenization of the components. The resulting solutions were loaded into syringes with industry grade 19G metallic needles and connected to a high-voltage power supply for electrospinning. A voltage of 20 kV was applied, and the electrospun mats were collected on an octagonal flat-plate collector lined with non-adherent paper (Fig. S1). The electrospinning conditions used in this study were adopted from a previous optimization study conducted by our research group, in which a factorial experimental design was used to systematically evaluate the effects of polymer concentration, applied voltage, flow rate, and tip-to-collector distance on the morphology of PVA electrospun fibers. The optimized conditions identified in that study were used as the basis for the present work (Libel et al. 2024).

All electrospinning equipment (high-voltage power supply, syringe pump, and collector) used in this study was manufactured by HSensor^®^ (Paraná, Brazil). Electrospinning was performed inside a laminar flow cabinet previously disinfected with 70% ethanol and exposed to UV light to maintain aseptic conditions.

### Characterization

#### Scanning and transmission electron microscopy

The morphology of the nanofibers was examined using scanning electron microscopy (SEM). Prior to imaging, the samples were sputter-coated with a thin gold layer to enhance their surface conductivity. SEM analyses were conducted using a Quanta 250 microscope (FEI, Thermo Fisher Scientific). The average fiber diameter was determined from SEM micrographs using ImageJ software by measuring 100 individual fibers at ×1500 magnification. The ultrastructural features of the PVA fibers and the internal structures associated with the incorporated fermentation broth were examined by transmission electron microscopy (TEM) using a JEOL JEM-1400 instrument (JEOL Ltd., Japan), with a nominal resolution of 0.2 nm and equipped with two CCD cameras for image acquisition. For TEM analysis, a thin layer of fibers was deposited directly onto carbon-coated copper grids (400 mesh) by electrospinning for approximately 30–60 s, following a previously reported approach (Braga et al. 2024).

#### Fourier-transform infrared spectroscopy-attenuated total reflectance (FTIR-ATR)

Fourier-transform infrared (FTIR) spectra were acquired using a PerkinElmer Spectrum spectrometer equipped with an attenuated total reflectance (ATR) accessory. Measurements were recorded in the range of 4000 –650 cm^− 1^, with a resolution of 4 cm^− 1^ and 32 scans. Spectra were obtained for the *B. amyloliquefaciens* fermentation broth, pure PVA, and electrospun fibers containing the encapsulated microorganism. For the fermented broth of *B. amyloliquefaciens*, an 50 µL aliquot was collected from the commercial flask, frozen, and lyophilized. Afterwards, the sample was dried under vacuum prior to analysis (Subramanian et al. 2007).

#### Mechanical assays

To evaluate the mechanical properties, PVA, PVA-BA50, and PVA-BA100 fibers were collected on a grounded rotating drum collector (12 cm diameter) operating at 300 rpm to improve the uniformity of fiber deposition and obtain homogeneous nanofibrous mats. For each formulation, 4 mL of spinning solution was electrospun to produce the specimens used for mechanical testing. The tensile properties of the polymeric fibers were measured by cutting samples into strips of 6.0 cm × 1.0 cm. The thickness was determined using a digital micrometer (0.001 mm resolution). Tensile tests were performed using a testing machine HSensor^*®*^(PR, Brazil) equipped with a 5 kg load cell. The tests were performed in triplicate with an initial gauge length of 5.0 cm at a constant elongation rate of 20 mm min^− 1^, based on ISO 527-3.

### Statistical analysis

Statistical analyses were performed to compare the average diameter measurements and mechanical properties of PVA fibers and PVA fibers containing *B. amyloliquefaciens* fermentation broth. Data were analyzed by one-way ANOVA followed by Tukey’s post hoc test, with significance set at *p* < 0.05, using GraphPad Prism 8.0.1.

### MALDI-TOF MS Identification

Microbial characterization of *B. amyloliquefaciens* was conducted using matrix-assisted laser desorption/ionization time-of-flight mass spectrometry (MALDI- TOF MS) in the linear positive ion mode. Fresh colonies (24 h on nutrient agar) were sampled with a loop, suspended in 300 µL ultrapure water, mixed with 900 µL absolute ethanol, vortexed, and centrifuged at 2046 ×g (4400 rpm) for 2 min. The pellet was air-dried, resuspended in 50 µL 70% formic acid and 50 µL acetonitrile, centrifuged at 2046 ×g (4400 rpm) for 2 min, and 1 µL of the supernatant was spotted on a MALDI plate and overlaid with 1 µL α-cyano-4-hydroxycinnamic acid (HCCA) matrix (10 mg/mL in 50% acetonitrile/2.5% trifluoroacetic acid). The samples were air-dried at room temperature prior to analysis. Mass spectra were acquired on a Microflex LT mass spectrometer (Bruker Daltonics) with laser intensity set between 65 and 70%, over a mass range of m/z 2,000–20,000. Each spectrum was obtained from 240 accumulated laser shots (6 positions × 40 shots) by applying a minimum intensity threshold of 500. The sample stage movement was randomized to ensure a uniform signal distribution. External calibration was performed using a standard peptide mix (Bruker Bacterial Test Standard or equivalent). Spectral data were processed using the MBT Compass software (Bruker) and matched against an in-house curated library of Bacillus reference strains. Peak lists were exported for further comparison, and reproducibility was confirmed across biological replicates.

### Biosurfactant Analysis by MALDI-TOF MS

The presence of biosurfactant-related compounds was evaluated by matrix-assisted laser desorption/ionization time-of-flight mass spectrometry (MALDI-TOF MS) operated in positive linear ion mode. Crude fermentation broth (50 mL) was acidified to pH 2.0 using 85% phosphoric acid and incubated overnight at 4 °C to promote lipopeptide precipitation. The acidified broth was subsequently centrifuged, and the resulting pellet was extracted with 300 µL of LC-MS-grade methanol by vortex mixing for 5 min. After centrifugation to remove insoluble material, the supernatant was used for MALDI-TOF MS analysis. An aliquot (1 µL) of the methanolic extract was mixed with 10 µL of α-cyano-4-hydroxycinnamic acid (HCCA) matrix solution (10 mg mL^− 1^ prepared in 50% acetonitrile containing 2.5% trifluoroacetic acid) directly on the MALDI target plate. Each sample was analyzed in triplicate by spotting onto three independent positions on the target plate and allowing the spots to air-dry at room temperature. Mass spectra were acquired using a Microflex LT mass spectrometer (Bruker Daltonics, Bremen, Germany) in positive linear mode over an m/z range of 600–2,000, using 240 accumulated laser shots (six positions × 40 laser shots). Spectra were processed using MBT Compass software, and peak profiles were evaluated for the presence of putative surfactin, iturin, and fengycin homologs. Because MALDI-TOF MS was used as a qualitative screening approach, the detected signals were considered putative assignments based on their characteristic *m/z* values and require confirmation by tandem mass spectrometry (MS/MS).

### Qualitative viability assessment

The viability of *B. amyloliquefaciens* was assessed by culturing the samples on TSA agar plates. Three conditions were evaluated: the bacterial fermentation broth, the polymeric spinning solution containing the fermentation broth, and electrospun nanofibers. To evaluate the stability of the electrospun nanofibers and their potential to preserve bacterial viability during storage, samples were stored for one year under ambient laboratory conditions in a sealed plastic container protected from light. These conditions were selected to approximate typical storage conditions for microbial formulations. Following the storage period, bacterial viability was assessed by evaluating the recovery of viable cells from the electrospun fibers. All tests were performed in triplicate. Following inoculation, the plates were incubated in a BOD chamber at 28 °C, and bacterial growth was monitored after 24, 48, and 72 h. Colonies obtained during incubation were further identified using MALDI-TOF MS to confirm the presence and identity of *B. amyloliquefaciens*.

### Comparative viability analysis

A comparative viability assessment was performed by serial dilution and plate counting on TSA agar. Aliquots of the *B. amyloliquefaciens* culture and the polymeric spinning solution containing *B. amyloliquefaciens* fermentation broth, as well as 1 cm^2^ fragments of electrospun fibers at the center of the electrospun material, were collected with a sterile inoculation loop and suspended in 0.9% saline containing 0.01% Tween 80, were vortexed, serially diluted up to 10^− 6^, and 100 µL aliquots were plated in triplicate. Plates were incubated at 28 °C for 24 h and the results were expressed as colony-forming units (CFU). For liquid samples, CFU values were expressed as CFU mL^− 1^ according to Eq. (1).

1 $$\:CFU\:=\:\frac{mean\:colony\:count\:\times\:\:dilution\:factor}{0.1\:mL}$$

For the electrospun fibers, viable cell counts were obtained from standardized 1 cm^2^ fiber fragments and are reported as CFU cm^− 2^. Because these measurements were obtained from standardized fragments of a heterogeneous nanofibrous material rather than from the entire electrospun mat, the results should be interpreted as comparative estimates of viable cell recovery rather than as absolute measures of bacterial concentration within the nanofibrous matrix.

## Results and discussion

### Morphological and chemical characterization

To investigate the feasibility of using electrospun nanofibers for stabilization of microbial products, a polymeric solution of PVA was successfully combined with *B. amyloliquefaciens* fermentation broth and electrospun into uniform nanofibers using a custom-built apparatus. Because the objective of the morphological, chemical, and mechanical characterization was to evaluate the effect of fermentation broth incorporation on the electrospun polymeric matrix, neat PVA fibers were used as the reference control for these analyses. Different formulations (PVA-BA50 and PVA-BA100) were evaluated to examine the effect of fermentation broth incorporation on fiber morphology. To the best of our knowledge, studies on *Bacillus* encapsulation generally do not consider the fermentation broth, focusing instead on *Bacillus* isolates subjected to freeze-drying. For this reason, concentrations were selected to explore the upper limit of the broth (100% v/v) and an intermediate condition (50% water and 50% fermentation broth), using a PVA solution prepared in water (100% v/v) as a reference.

**Fig. 1:**
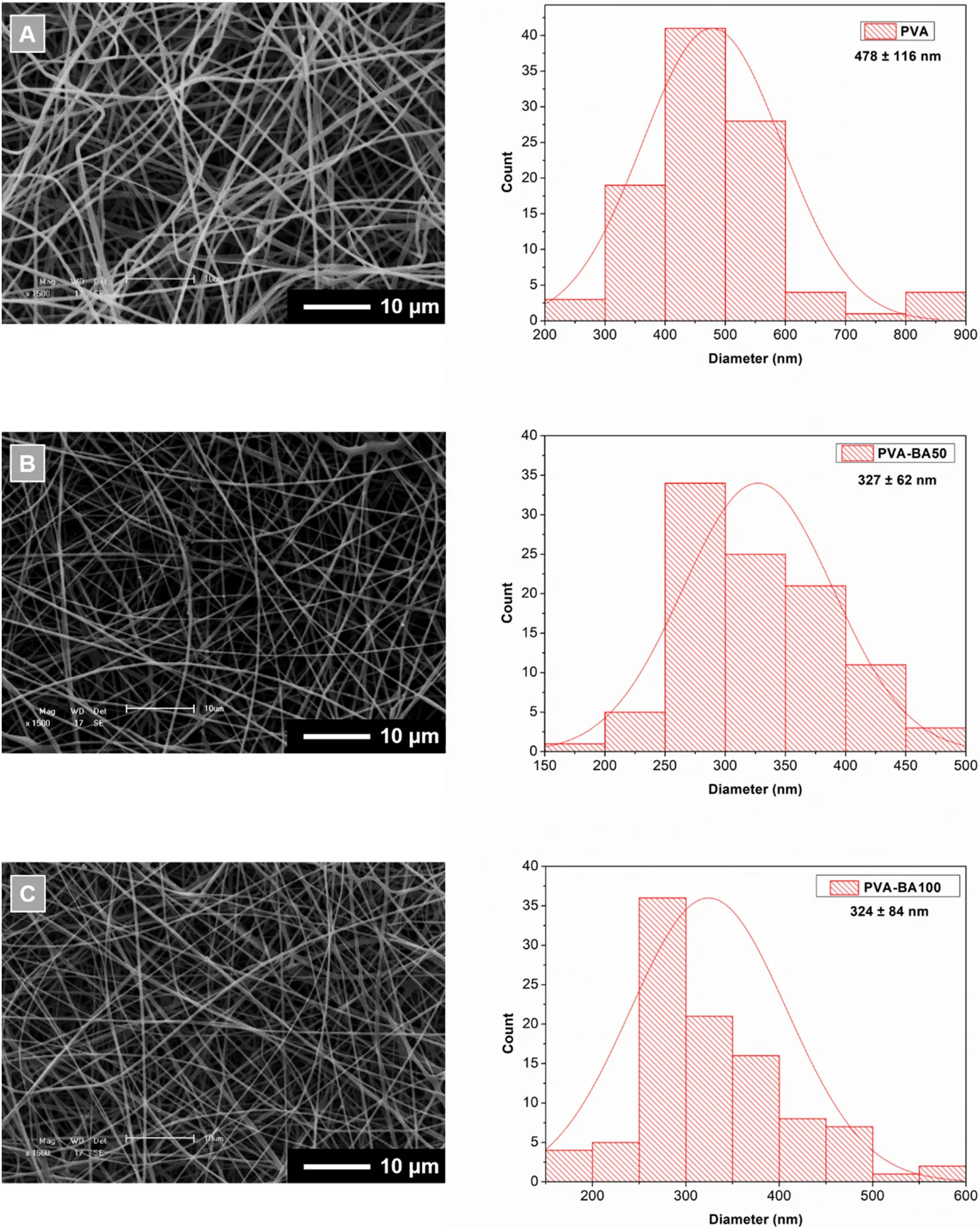
SEM images of (**A**) PVA, (**B**) PVA-BA50, and (**C**) PVA-BA100 fibers, with their respective average diameter distribution histograms and standard deviation. Quantitative measurements were performed using ImageJ software by analyzing 100 fiber diameters per SEM image (magnification ×1500, scale bar 10 μm)

SEM analysis revealed that the pure PVA fibers exhibited a continuous and uniform morphology, with an average diameter of 478 ± 116 nm. Upon incorporating the *B. amyloliquefaciens* fermentation broth, the average fiber diameter decreased significantly to 327 ± 62 nm for PVA-BA50 and 324 ± 84 nm for PVA-BA100, and the distribution became narrower, resulting in more homogeneous nanofiber mats (Fig. 1). This morphological refinement may be associated with the presence of biosurfactants in the fermentation broth, which can reduce the surface tension of the electrospinning solution and promote better molecular alignment of the polymer chains during jet elongation (Oldal et al. 2023). In addition to reducing surface tension, biosurfactants may also influence other physicochemical properties of the spinning solution, including viscosity, electrical conductivity, and surface charge, which are known to affect jet stability, polymer stretching, solvent evaporation, and ultimately the diameter and uniformity of electrospun fibers (Hu et al. 2015; Naeim et al. 2025). Although these physicochemical properties were not directly measured in the present study, the observed reduction in fiber diameter and the narrower diameter distribution are consistent with these well-established electrospinning mechanisms. This interpretation is further supported by the statistical analysis of the fiber diameters.

The statistical analysis (ANOVA) indicated that although fibers containing *B. amyloliquefaciens* broth at 50% and 100% (v/v) showed a trend of decreasing diameter, the differences between these two concentrations were not statistically significant (*p* > 0.05) (Fig. S2). This may indicate a saturation effect in the biosurfactant-mediated reduction of surface tension, supporting the conclusion that the incorporation of microbial metabolites was sufficient to produce more uniform fiber morphology.

**Fig. 2:**
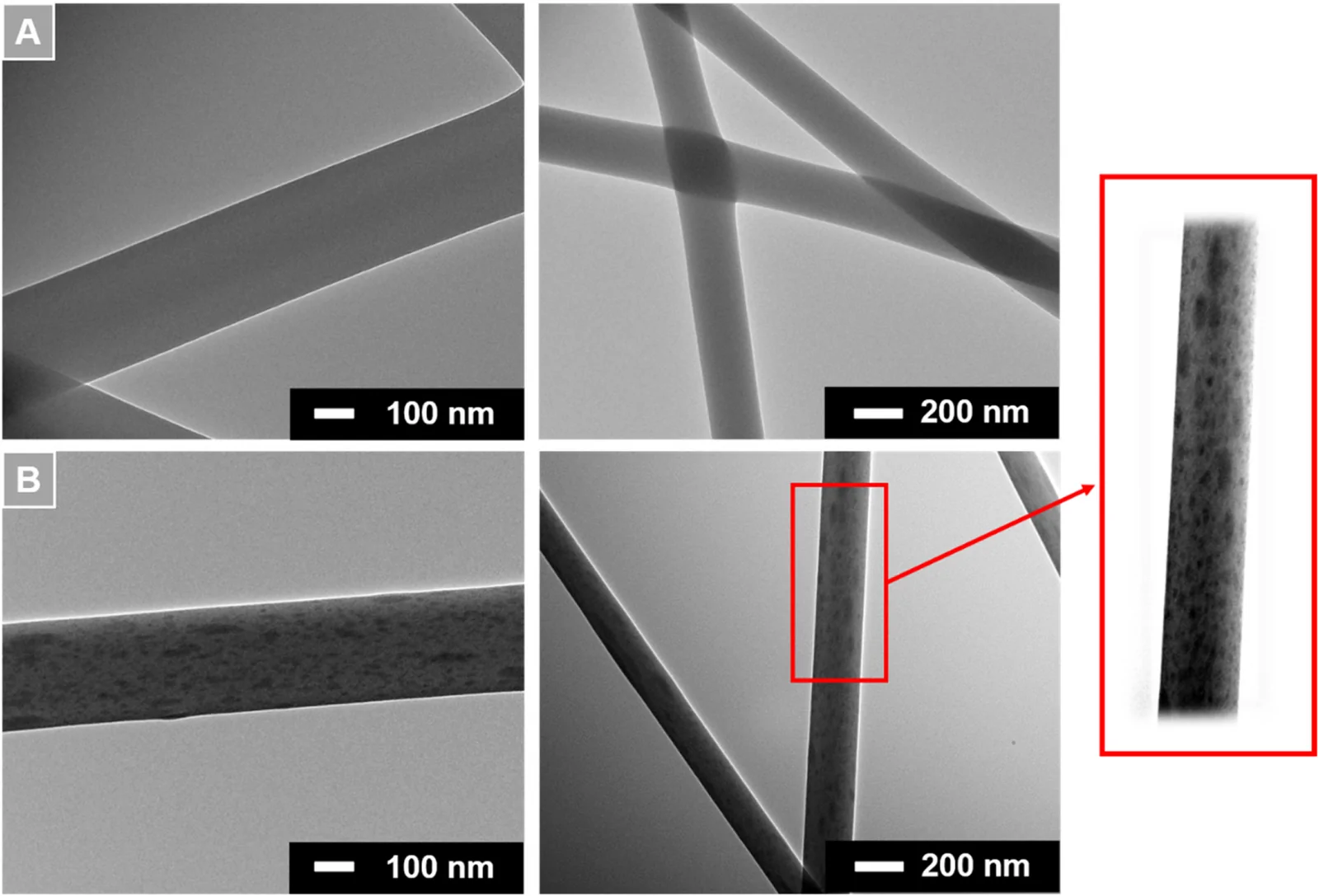
TEM images of (**A**) PVA fibers, and (**B**) PVA-BA100 fibers containing the fermented broth of *B. amyloliquefaciens*

TEM revealed electron-dense microdomains distributed throughout the PVA-BA100 fibers that were absent in the neat PVA fibers (Fig. 2). The internal fiber structure remained dense and continuous, indicating that incorporation of the fermentation broth did not compromise fiber integrity. These electron-dense regions are consistent with the incorporation of fermentation broth components into the polymer matrix and complement the SEM observations, which showed no bacterial cells exposed on the fiber surface.

**Fig. 3:**
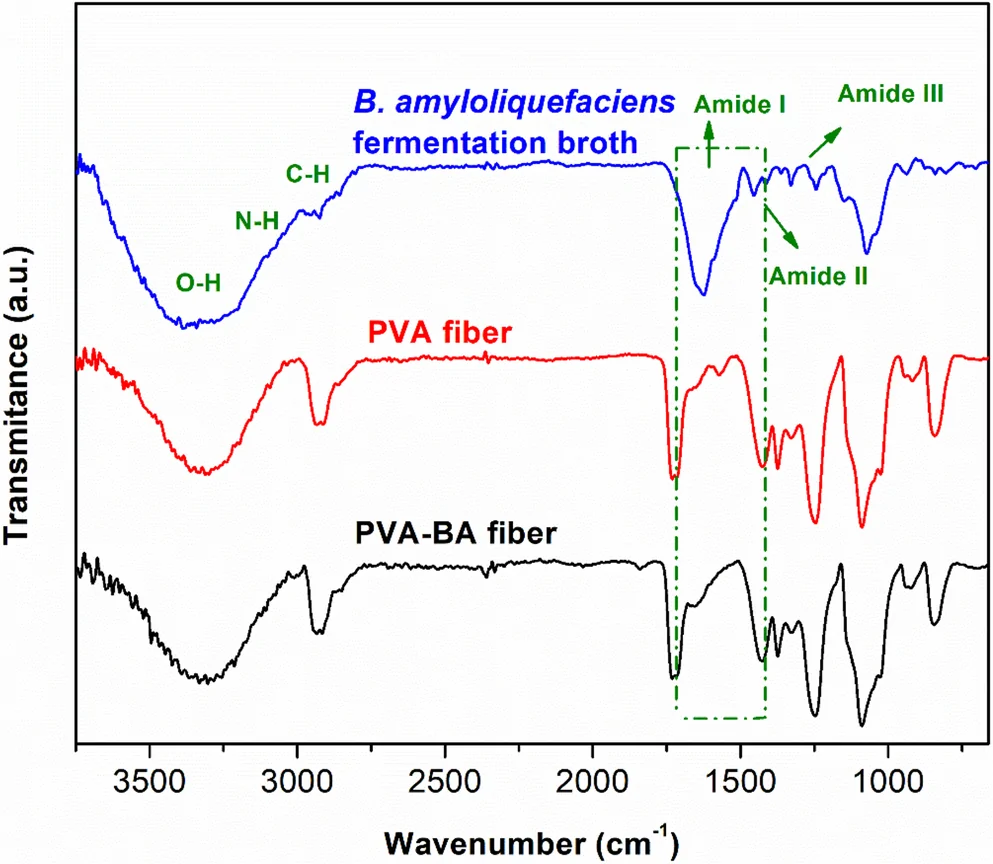
FTIR-ATR spectra of *B. amyloliquefaciens* fermented broth, PVA fiber, and PVA/BA fibers

The FTIR-ATR spectra of the PVA and PVA/*B. amyloliquefaciens* nanofibers exhibited the characteristic absorption bands of PVA at approximately 3300 cm^− 1^ (O–H stretching), 2940 cm^− 1^ (C–H stretching), and 1090 cm^− 1^ (C–O stretching of the secondary alcohol groups), together with additional bands at 1726, 1245, 1144, and 1020 cm^− 1^ that are characteristic of residual acetate groups and the polymer backbone (Fig. 3) (Sánchez-León et al. 2023). Other signals attributed to the residual acetate fraction in PVA are observed at 1726 cm^− 1^, corresponding to C = O stretching; at 1245 cm^− 1^, related to C-O-C ester groups; at 1144 cm^− 1^, associated with the C-O stretching of the hydrolyzed fraction and with crystallinity; and at 1020 cm^− 1^, corresponding to the C-O stretching of the main chain (Han et al. 2015). Upon the incorporation of the Bacillus fermentation broth, new bands appeared at 1650 and 1540 cm^− 1^, corresponding to amide I and amide II vibrations, respectively (Fatima and Singh 2022).

These peaks indicate the presence of proteinaceous and lipid-derived compounds derived from the fermentation broth, suggesting hydrogen bonding and secondary interactions between the polymer chains and biological components. The spectral shifts support the interpretation that microbial metabolites were successfully integrated into the polymer matrix, which may have contributed to the enhanced mechanical and structural stability.

### Mechanical properties

**Fig. 4:**
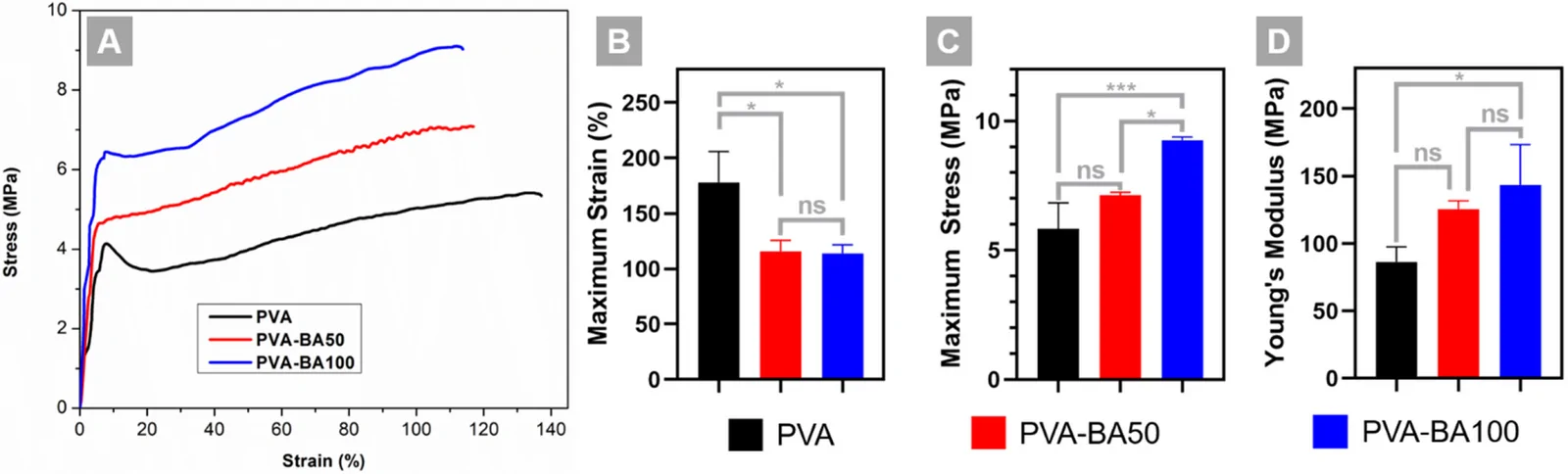
Typical stress-strain curve for PVA, PVA-BA50 and PVA-BA100 fibers (**A**); ANOVA statistical analysis for the mechanical properties of maximum strain (**B**), maximum stress (**C**) and Young’s modulus (**D**). The terms * and *** indicate a statistical difference between the samples with a minimum confidence level of *p* ≤ 0.05 and *p* ≤ 0.001, respectively. The term ns means that the results are not statistically different from each other (*p* > 0.05). Mechanical tests were performed in triplicate (*n* = 3), and error bars indicate the standard deviation for each sample

Mechanical testing results showed a significant increase in tensile strength and Young’s modulus with the addition of microbial broth, accompanied by a moderate reduction in elongation at break (Fig. 4; Table 1). PVA mats exhibited a maximum deformation of 177 ± 23%, tensile strength of 5.8 ± 0.9 MPa, and Young’s modulus of 86 ± 9 MPa. The incorporation of 50% and 100% (v/v) of *B. amyloliquefaciens* broth increased the tensile strength to 7.2 ± 0.1 and 9.3 ± 0.1 MPa, respectively, and the modulus to 125 ± 5 and 143 ± 24 MPa, while reducing the elongation to approximately 115%. These results indicate that the microbial metabolites may have acted as natural reinforcing agents, improving fiber orientation and interchain cohesion.

**Table 1:** Maximum strain, maximum stress, and Young’s modulus values for the samples

Sample	Maximum Strain (%)	Maximum Stress (MPa)	Young’s Modulus (MPa)
PVA	177 ± 23	5.8 ± 0.9	86 ± 9
PVA-BA50	116 ± 8	7.2 ± 0.1	125 ± 5
PVA-BA100	114 ± 6	9.3 ± 0.1	143 ± 24

Such behavior is consistent with surfactant-induced stabilization mechanisms previously reported for emulsion electrospinning systems, in which reductions in surface tension facilitate enhanced jet stretching and polymer chain alignment along the fiber axis (Hu et al. 2015). This process leads to denser and more compact nanofibrous mats with fewer structural defects, resulting in increased stiffness and mechanical strength. These structural features may be advantageous for maintaining fiber integrity during storage and subsequent handling of the microbial formulation.

### Viability and stability of B. amyloliquefaciens after electrospinning

**Fig. 5:**
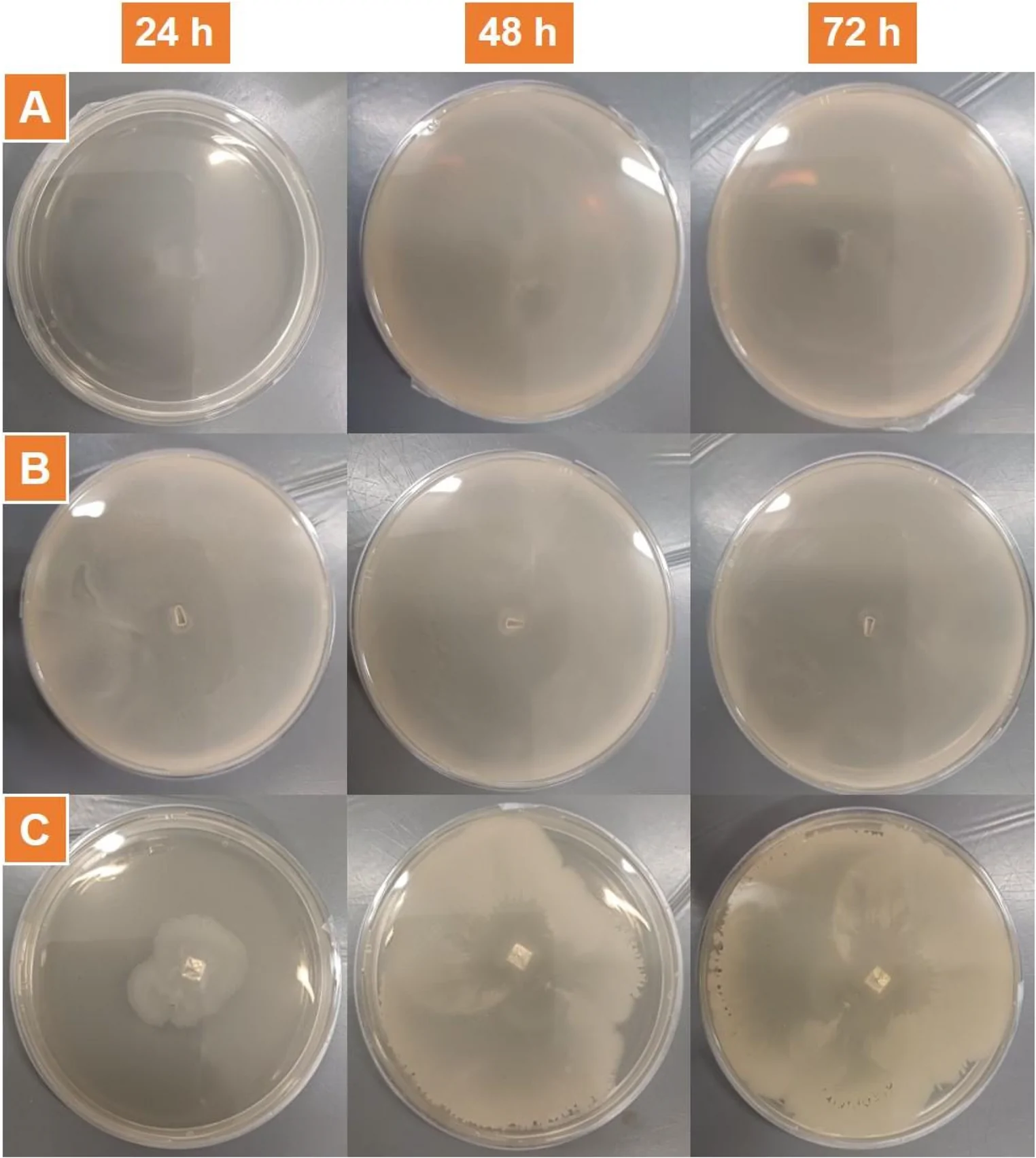
Bacterial growth from (**a**) fermented broth, (**b**) polymeric solution containing the fermented broth, and (**c**) electrospun fibers containing the fermented broth was monitored after 24, 48, and 72 h

The viability of bacteria encapsulated in electrospun fibers was assessed by culturing the fibers on TSA agar plates and comparing the bacterial growth with samples directly inoculated from the fermented broth or polymeric spinning solution without electrospinning. In all cases, bacterial growth was observed at varying rates. The fastest growth occurred with cultures from the fermented broth, followed by the polymeric solution, and finally the electrospun fibers (Fig. 5). These differences in growth rates are expected because bacterial cells encapsulated within the electrospun matrix must overcome additional physical barriers before proliferation. Nevertheless, the observed retention of viable colony formation indicates that bacterial viability was maintained despite the encapsulation process. These observations demonstrate the recovery of viable cells following electrospinning, although they do not directly assess the agricultural performance of the formulation.

Analysis of viable cell recovery further confirmed the survival of *B. amyloliquefaciens* after electrospinning. Viable cells were recovered from the *B. amyloliquefaciens* culture, the polymeric pre-spinning solution, and the electrospun fibers (Table 2). Because the electrospun mats represent a heterogeneous solid material, viable counts obtained from standardized 1 cm^2^ fiber fragments are presented as comparative estimates of viable cell recovery (CFU cm^− 2^) rather than as absolute volumetric concentrations. The results indicate that viable cell recovery decreased after incorporation of the fermentation broth into the polymeric pre-spinning solution and decreased further after electrospinning. This reduction suggests that the formulation process-imposed stress on part of the bacterial population, resulting in a partial loss of recoverable cells. Nevertheless, the recovery of viable cells after electrospinning demonstrates that a substantial fraction of the bacterial population remained viable throughout the electrospinning process and following incorporation into the PVA nanofibers.

**Table 2:** Comparative assessment of *B. amyloliquefaciens* recovery before and after electrospinning

Sample	Mean colony count	CFU	log_10_ CFU
Isolated *B. amyloliquefaciens* culture	189.33	1.89 × 10^9^ (CFU mL^− 1^)	9.27
Polymeric spinning solution	8.33	8.33 × 10^7^ (CFU mL^− 1^)	7.92
Electrospun fibers	3.33	3.33 × 10^7^ (CFU cm^− 2^)	7.52

Electrospinning uses an electric field to generate polymeric fibers from a polymer solution (Diep and Schiffman 2023). The recovery of viable cells after electrospinning is likely associated with multiple complementary factors rather than a single protective mechanism. *Bacillus* species are capable of forming endospores under unfavorable environmental conditions, and these specialized structures possess multiple protective layers that confer resistance to dehydration and other physical stresses (Herrmann et al. 2024). Although endospore formation was not directly evaluated in the present study, the presence of endospores may have contributed to bacterial survival during electrospinning and storage. Consistent with this hypothesis, the resilience of *Bacillus* species during electrospinning has been highlighted in recent literature, where the intrinsic resistance of spores was proposed as an important factor contributing to the maintenance of microbial viability throughout the high-voltage process (Vehapi et al. 2024). In addition, *Bacillus* spp. are known to produce extracellular polymeric substances and secreted proteins during growth (Beauregard et al. 2013). Although these extracellular components were not specifically characterized in this study, compounds naturally present in the fermentation broth may have contributed to the formation of a protective microenvironment during fiber formation and drying. The use of an aqueous PVA solution may also have favored cell preservation, as PVA is a hydrophilic and biocompatible polymer processed without toxic organic solvents (Deng and Zhang 2020). Furthermore, encapsulation within the polymeric matrix may have reduced cellular dehydration and minimized environmental fluctuations during storage, which is consistent with the recovery of viable cells after prolonged storage.

**Fig. 6:**
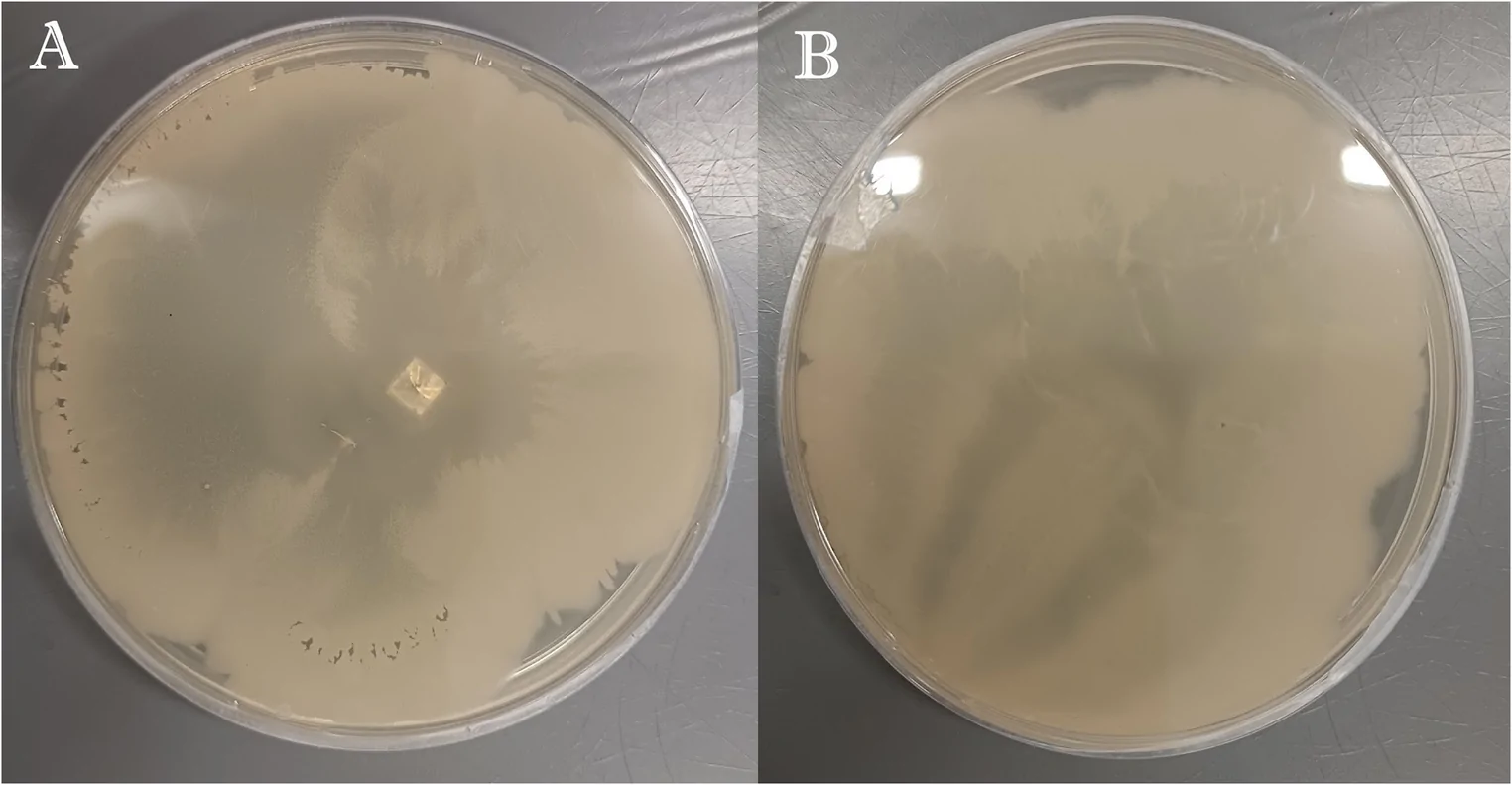
Representative photographs of TSA plates showing bacterial outgrowth from electrospun fiber fragments containing *B. amyloliquefaciens* fermentation broth. (**A**) Fiber fragments after electrospinning and 72 h incubation. (**B**) Fiber fragments after one year of ambient storage followed by 72 h incubation. Bacterial growth originated directly from the electrospun fiber fragments rather than from conventional serial dilution plating

In addition to confirming the identity of *B. amyloliquefaciens*, the electrospun formulation demonstrated the recovery of viable cells after one year of storage under ambient conditions (Fig. 6). However, the experimental design did not include intermediate monitoring time points or a non-encapsulated control stored under identical conditions, limiting the present study to a proof-of-concept demonstration. Future investigations should include systematic viability assessments at multiple storage intervals and direct comparisons with non-encapsulated formulations to quantify the protective contribution of the PVA matrix to microbial preservation.

**Fig. 7:**
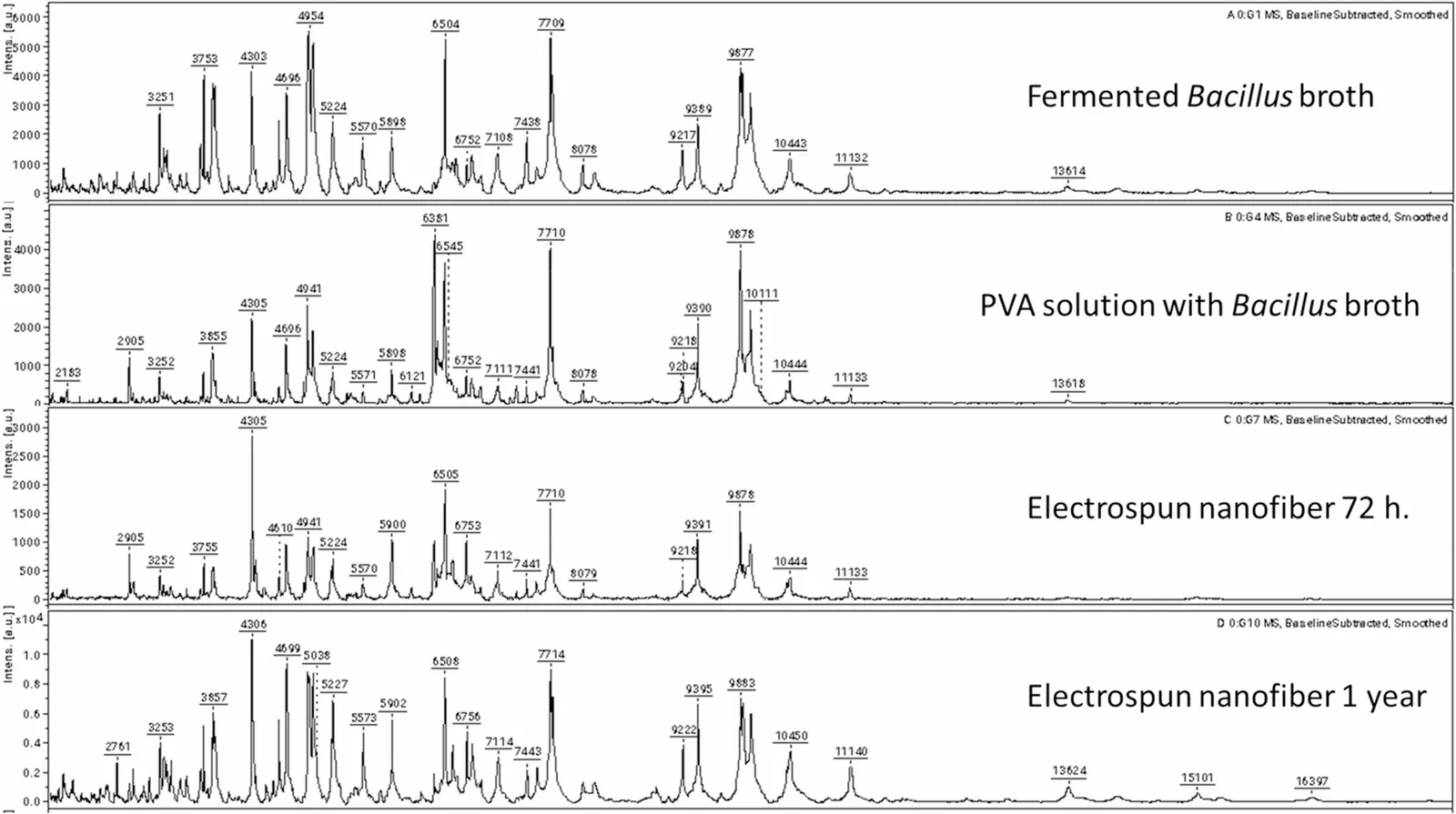
MALDI-TOF MS spectra of the fermentation *B. amyloliquefaciens* broth, PVA solution with *B. amyloliquefaciens* broth, electrospun nanofiber after 72 h, and electrospun nanofiber after 1 year obtained to identify the Bacillus species

MALDI-TOF MS identified the recovered isolates within the *Bacillus subtilis* species complex, with the closest matches corresponding mainly to *Bacillus amyloliquefaciens* and *Bacillus velezensis* (Fig. 7; Table S1). This result was expected, as both species are part of the *Bacillus subtilis* species complex and share highly similar proteomic and genomic profiles (Dunlap et al. 2016). The overlap in MALDI-TOF signatures has been reported previously and does not affect the functional interpretation of our results, since both taxa are well recognized as plant growth-promoting rhizobacteria with biocontrol activity (Fan et al. 2017).

### Detection of Biosurfactant Lipopeptides by MALDI-TOF

MALDI-TOF MS analysis of the fermented *B. amyloliquefaciens* broth confirmed the strain identity and revealed the presence of molecular ions consistent with the presence of cyclic lipopeptides. Signals detected in the *m/z* range of approximately 1,020–1,094 were consistent with the mass range reported for surfactin and iturin homologs, whereas ions detected between *m/z* 1,435–1,548 were consistent with fengycin homologs (Fig. S3) (Yang et al. 2015). Because of the partial overlap between surfactin and iturin homologs, together with the inherent limitations of linear MALDI-TOF MS, these assignments should be regarded as putative and interpreted with appropriate caution. These lipopeptides, including surfactins, iturins, and fengycins, are non-ribosomal secondary metabolites widely produced by *Bacillus* spp. and are well recognized for their biocontrol activities (Schalchli et al. 2023). They exert broad-spectrum antagonistic effects by disrupting pathogen cell membranes, inducing systemic resistance in plants, and enhancing microbial competitiveness within ecological niches (Kamath and Patel 2025).

The putative presence of these microbial metabolites in the fermentation broth may have contributed to the formation of electrospun nanofibers with controlled morphology and improved mechanical properties. SEM and TEM analyses revealed uniform fiber diameters and continuous structures, while FTIR spectra confirmed the incorporation of microbial metabolites into the polymer matrix. Mechanical testing showed increased tensile strength and Young’s modulus compared to PVA fibers without the fermentation extract, indicating improved resilience and structural integrity.

Viability assays demonstrated that *B. amyloliquefaciens* cells remained alive post-electrospinning, supporting the potential use of the nanofibers as microbial carriers. The recovery of viable cells is consistent with a potential protective role of the PVA matrix, suggesting that electrospun fibers can serve as effective carriers for beneficial microbes. These results indicate that electrospun PVA nanofibers can support microbial viability while maintaining suitable structural properties for formulation purposes. Such formulations may contribute to improved stabilization and handling of beneficial microorganisms.

Stability assessments were conducted under controlled laboratory conditions and should therefore be considered a proof-of-concept for the use of electrospun PVA nanofibers as a microbial stabilization strategy. Agricultural environments involve complex interactions with soil microbiota, fluctuating temperature and humidity, UV exposure, and other environmental factors that were not evaluated in the present study. Therefore, the potential agronomic applications of the electrospun formulation, including seed coating and soil delivery of beneficial microorganisms, require validation under greenhouse and field conditions. Future studies should directly compare electrospun and liquid fermentation formulations by assessing bacterial survival, release dynamics, root colonization, plant growth promotion, and biocontrol efficacy. Additionally, the influence of PVA concentration on bacterial release kinetics, matrix dissolution, and overall formulation performance warrants further investigation.

## Conclusion

This study demonstrates the feasibility of directly incorporating *Bacillus amyloliquefaciens* fermentation broth into electrospun PVA nanofibers while preserving bacterial viability, without requiring prior cell isolation, washing, or lyophilization. Morphological, ultrastructural, spectroscopic, mechanical, and microbiological analyses confirmed the successful incorporation of the fermentation broth into the polymeric matrix and the recovery of viable bacterial cells following electrospinning. Importantly, viable cells remained recoverable after one year of storage under ambient conditions, demonstrating bacterial viability after the storage period evaluated.

Beyond demonstrating microbial survival after electrospinning, this work introduces a simplified formulation strategy based on the direct electrospinning of crude fermentation broth, avoiding conventional downstream purification procedures that are commonly required before encapsulation. Although direct comparisons with non-encapsulated formulations and validation under greenhouse and field conditions are still necessary, the present study provides a proof-of-concept that supports the potential of electrospinning as a practical platform for microbial formulation. By simplifying the formulation process while maintaining bacterial viability after prolonged storage, this approach may contribute to the development of more efficient, scalable, and practical microbial products for future agricultural applications.

## Supplementary Information

Below is the link to the electronic supplementary material.

Supplementary Material 1 (DOCX 1.90 MB)

## Data Availability

No datasets were generated or analysed during the current study.
