# Supplementary material for: Electrospun PVA nanofibers improve the storage stability of Bacillus amyloliquefaciens fermentation broth
Source: World J Microbiol Biotechnol. 2026 Oct 9;42(10):597. doi: 10.1007/s11274-026-05334-w (PMC13652878; doi:10.1007/s11274-026-05334-w)
Supplement: Supplementary file 1 [file 11274_2026_5334_MOESM1_ESM.docx]

Supporting Information for

**Electrospun PVA nanofibers improve the storage stability of *Bacillus amyloliquefaciens* fermentation broth**

Paula Derksen Macruz^1#^, Giovanna Picoli Libel^1#^, Lucas Haruo Nakayama Pereira^2^, Juliano Cesar da Silva^3^, Sergio Antônio Barraca^3^, Eduardo Radovanovic^2^, Eduardo Jorge Pilau^1, 2*^

^1^ State University of Maringá (UEM), Department of Chemical, Maringá, Paraná, Brazil

^2^ State University of Maringá (UEM), Postgraduate Program in Biological Sciences, Paraná, Brazil

^3^ Ballagro Agro Tecnologia, Sumaré, SP, Brazil

*Corresponding author: pg55847@uem.br

**^#^**These authors contributed equally

**Figure S1.** Schematic illustration of the electrospinning setup, consisting of a high-voltage power supply, a syringe pump for controlled solution delivery, and an octagonal flat-plate collector for fiber deposition.

**Figure S2.** Statistical ANOVA analysis regarding the calculated mean diameter for PVA, PVA-BA50, and PVA-BA100 fibers. The term **** indicate a statistical difference between the samples with a minimum confidence level of p ≤ 0.0001. The term *ns* means that the results are not statistically different from each other (p > 0.05).

**Table S1.** Identification of *Bacillus* species in fermented broth, PVA solution and electrospun nanofibers using MALDI-TOF MS.

| **Analyte Name** | **Organism (best match)** | **Score Value *** | **Median Score Value** | **Result** |
| --- | --- | --- | --- | --- |
| Fermented *Bacillus* broth | *Bacillus velezensis* | 2,37 | ***2,42*** | *Bacillus velezensis* |
| Fermented *Bacillus* broth | *Bacillus velezensis* | 2,50 |  |  |
| Fermented *Bacillus* broth | *Bacillus velezensis* | 2,38 |  |  |
| PVA solution with *Bacillus* broth | *Bacillus amyloliquefaciens* | 2,28 | ***2,28*** | *Bacillus amyloliquefaciens* |
| PVA solution with *Bacillus* broth | *Bacillus amyloliquefaciens* | 2,32 |  |  |
| PVA solution with *Bacillus* broth | *Bacillus amyloliquefaciens* | 2,25 |  |  |
| Electrospun nanofibers 72 hours | *Bacillus amyloliquefaciens* | 2,28 | ***2,29*** | *Bacillus amyloliquefaciens* |
| Electrospun nanofibers 72 hours | *Bacillus velezensis* | 2,28 |  |  |
| Electrospun nanofibers 72 hours | *Bacillus subtilis* | 2,30 |  |  |
| Electrospun nanofibers 1 year | *Bacillus velezensis* | 2,40 | ***2,37*** | *Bacillus velezensis* |
| Electrospun nanofibers 1 year | *Bacillus velezensis* | 2,35 |  |  |
| Electrospun nanofibers 1 year | *Bacillus velezensis* | 2,36 |  |  |

* Range of Score Values: 3.00 - 2.30: highly probable species identification; 2.29 - 2.00: secure genus identification, probable species identification; 1.99 - 1.70: probable genus identification; 1.69 - 0.00: not reliable identification.

**Figure S3.** MALDI-TOF MS spectrum of the fermentation extract from *B. amyloliquefaciens*. Molecular ions in the *m/z* range of 1,006–1,060 are attributed to putative surfactin homologs, ions in the range of 1,060–1,283 correspond to iturin homologs, and ions in the range of 1,433–1,507 correspond to fengycin homologs.
